# Real-World Treatment Patterns, Health Outcomes, and Healthcare Resource Use in Advanced Common EGFR-Positive Non-Small Cell Lung Cancer Patients Treated with Osimertinib in Alberta

**DOI:** 10.3390/curroncol31080327

**Published:** 2024-07-31

**Authors:** Winson Y. Cheung, Chantelle Carbonell, Vishal Navani, Randeep S. Sangha, Emmanuel M. Ewara, Julia Elia-Pacitti, Sandra Iczkovitz, Tamer N. Jarada, Matthew T. Warkentin

**Affiliations:** 1 Department of Oncology, Cumming School of Medicine, University of Calgary, Calgary, AB T2N 1N4, Canadatnjarada@ucalgary.ca (T.N.J.);; 2 Department of Oncology, Faculty of Medicine & Dentistry, University of Alberta, Edmonton, AB T6G 2R3, Canada; 3 Johnson & Johnson Innovative Medicine, Toronto, ON M3C 1L9, Canada

**Keywords:** osimertinib, non-small cell lung cancer, advanced disease, treatment, outcomes

## Abstract

There is limited information on the treatment trajectory and outcomes of patients with advanced cEGFRm NSCLC treated with osimertinib in routine clinical practice in Canada. By using and analyzing population-based administrative data and detailed chart abstraction in the province of Alberta, our objective was to capture Canadian-specific real-world treatment patterns, health outcomes, and healthcare resource utilization (HCRU) in advanced cEGFRm NSCLC patients who were (a) treated with osimertinib and (b) those receiving treatment after osimertinib. In our study cohort, we found that the overall survival rates for real-world patients receiving osimertinib were less favorable than those observed in clinical trials (24.0 versus 38.6 months). The attrition rate after osimertinib was substantial and high HCRU persisted across many years after diagnosis and treatment. This study provides important real-world evidence on contemporary survival, treatment patterns, and healthcare use among cEGFRm NSCLC patients treated with osimertinib and suggests that further research efforts are needed to improve therapeutic options in both the first and subsequent line settings.

## 1. Introduction

Advances in the treatment of lung cancer and smoking cessation strategies have contributed to significant decreases in the lung cancer incidence and mortality rates in Canada over the last decade [1]. Despite these advances, lung cancer continues to be the most commonly diagnosed cancer and the leading cause of cancer-related deaths in the country [1]. It is estimated that 31,000 Canadians were diagnosed with a lung cancer in 2023 (15,300 in men; 15,800 in women), and that 1 in 14 Canadians will be diagnosed with the disease in their lifetime [1]. The prognosis for patients diagnosed with an incident lung cancer continues to be challenging as well. Lung cancer accounts for approximately 1 in 4 cancer-related deaths in Canada, and 5-year net survival estimates for patients diagnosed with locally advanced (stage III) or metastatic (stage IV) lung cancers are only 15.3% and 3.1%, respectively [1].

Approximately 88% of newly diagnosed lung cancer cases in Canada are non-small cell lung cancers (NSCLC), of which approximately 15% harbor a mutation in the epidermal growth factor receptor (EGFR) gene [2,3,4,5,6]. Mutations in the EGFR gene typically occur in Exons 18 to 21 encoding a portion of the EGFR kinase domain. Approximately 90% of these mutations are exon 19 deletions and L858R point mutations on exon 21 [7], which are referred to as common EGFR mutations (cEGFRm) [8]. The remaining 10% of EGFR mutations are made up of uncommon mutations, including exon 20 insertions [7,9,10]. Several oral targeted therapies directed at the tyrosine kinase inhibitor (TKI) domain of the EGFR gene have demonstrated superior efficacy in comparison with cytotoxic chemotherapy and these TKIs have transformed the standard of care for the treatment of advanced cEGFRm NSCLC.

In Canada, the current recommended first-line (1L) treatment for advanced cEGFRm NSCLC is an EGFR TKI, with osimertinib (3rd generation TKI) preferred over 1st generation (gefitinib, erlotinib) and 2nd generation (afatinib) EGFR TKIs based on its superior overall survival benefit vs. comparator EGFR TKIs in the Phase III FLAURA trial and vs. platinum therapy plus pemetrexed in the Phase III AURA3 trial [11,12,13]. Osimertinib is active in patients with central nervous system (CNS) metastases and is also effective in the treatment of T790M mutations, which commonly develop when resistance to a 1st- or 2nd- generation EGFR TKI emerges [3,4,14]. The MARIPOSA-2 trial regimen amivantamab plus chemotherapy is a potential second-line treatment option [15]. Treatment strategies in the post-osimertinib setting are shaped by the line of therapy in which osimertinib was initially received. For example, in patients treated with 1L osimertinib, the current recommended second-line (2L) treatment (i.e., post-osimertinib setting) is platinum-based doublet chemotherapy, followed by docetaxel as a third-line (3L) option, or enrollment in a clinical trial [5,16], whereas for the subset of T790M-mutation positive patients treated in the 1L setting with either a 1st or 2nd generation EGFR TKI (afatinib, gefitinib, or erlotinib), the recommended 2L treatment is osimertinib followed by platinum-based doublet chemotherapy (3L) and then docetaxel as a subsequent line of therapy [5,16].

While the introduction of targeted oral EGFR TKIs has improved outcomes in patients with advanced cEGFRm NSCLC, treatment of this patient population remains challenging due to the development of resistance mutations. First- and second-generation EGFR TKIs acquire resistance mainly from T790M co-mutation, whereas resistance mechanisms to osimertinib are more varied, involving on-target mutations or off-target bypass tracts. This complex mutational landscape poses a significant clinical challenge [17,18,19,20,21,22]. Emerging 1L treatment strategies that aim to improve overall outcomes in patients with advanced cEGFRm NSCLC include osimertinib in combination with platinum-based doublet chemotherapy, and the bispecific EGFR and MET antibody, amivantamab, in combination with the 3rd generation EGFR TKI, lazertinib [11,23]. Even with these options, the challenges described post-osimertinib remain.

Unfortunately, there is limited information on outcomes in patients with advanced cEGFRm NSCLC treated with osimertinib as part of routine clinical practice in Canada. To address this gap, the objective of this study was to capture Canadian-specific real-world treatment patterns, health outcomes, and healthcare resource utilization (HCRU) in advanced cEGFRm NSCLC patients who were (a) treated with osimertinib (overall cohort) and (b) those receiving treatment after osimertinib (post-osimertinib cohort).

## 2. Materials and Methods

### 2.1. Study Population and Data Sources

Data for this study were obtained from administrative sources and detailed chart abstractions from electronic medical records (EMR). The Alberta Cancer Registry (ACR), a databank that captures more than 99% of cancers in the province of Alberta, Canada, was used to identify all individuals 18 years or older who were diagnosed with NSCLC between 2013 and 2020. The ACR data included patient, cancer, and treatment-related information. Somatic genomic sequencing data were used to identify those who tested positive for a cEGFRm, defined as those with an Exon 19 deletion or L858R mutation. Systemic therapy data from the Pharmaceutical Information Network (PIN) were used to identify those treated with osimertinib.

An administrative data algorithm was implemented to define lines of systemic therapy as follows: (A) the initial systemic therapy regimen was classified based on all systemic agents received within 30 days of initiating the first agent on or after the date of diagnosis and (B) initiation of any subsequent line of therapy was defined as the earliest of the following two events: (1) receipt of any new systemic agents not within the initial regimen (to capture a switch to a new therapy); or (2) a gap of more than 180 days between successive treatment dispensations (to capture re-treatment with the same therapy). These definitions align with those commonly used in the oncology real-world evidence literature [24,25,26].

For cEGFRm NSCLC diagnosed after 2020, ACR data did not exist. Therefore, we used data from the PIN to identify all patients treated with osimertinib. We performed detailed chart abstractions for this cohort to confirm a cEGFRm NSCLC diagnosis, and then collected patient, cancer, and treatment-related information by reviewing the Cancer Centre Electronic Medical Record (ARIA MO). HCRU data were obtained from the Discharge Abstract Database (DAD) and the National Ambulatory Care Reporting System (NACRS) and linked to the ACR and chart data using a unique lifetime identifier given to all residents of Alberta. We conducted an updated chart review for all participants who were still alive at the time of the latest chart abstraction (chart participants) or at the end of follow-up (administrative participants). For each patient (N = 202), we reviewed charts to identify the updated vital status and treatment information. The last date of follow-up from the chart review was 31 May 2023. Our study had two cohorts of interest: (1) patients treated with osimertinib at any line (referred to as the overall cohort) and (2) patients who were treated with a subsequent therapy after osimertinib (referred to as the post-osimertinib cohort).

### 2.2. Statistical Analyses

Patient characteristics were reported and stratified by cohort membership (overall and post-osimertinib). Means and standard deviations (SD) were reported for continuous variables and frequencies and proportions were reported for categorical variables. Missing data were summarized for all variables for descriptive purposes, but only complete case analyses were performed for time-to-event endpoints. Outcomes of interest for this study were overall survival (OS) and time-to-next-treatment or death (TTNTD). OS was defined as the time from the index date until the date of death (from any cause) or censoring at the last known date of contact or at the end of the study’s observation follow-up window. TTNTD was defined as the time from the index date until the initiation of a subsequent line of therapy, death (from any cause), or censoring at the last known date of contact or the end of the study observation follow-up window. The index date was either: (1) the initiation date of a given line of therapy for the overall cohort, or (2) the initiation date of the subsequent line of therapy received after osimertinib for the post-osimertinib cohort. We estimated survival curves for OS and TTNTD using the Kaplan–Meier (i.e., product-limit) estimator. We reported the median survival and survival at fixed time horizons (e.g., 1-year, 2-year, etc.) with 95% confidence intervals (CI). We also reported HCRU (e.g., hospitalizations) for up to five years of follow-up from the index date. Subgroup analyses were performed to report outcomes based on key factors. All statistical analyses were performed using R version 4.2.1 (2022-06-23).

## 3. Results

### 3.1. Patient Characteristics

**Figure 1:**
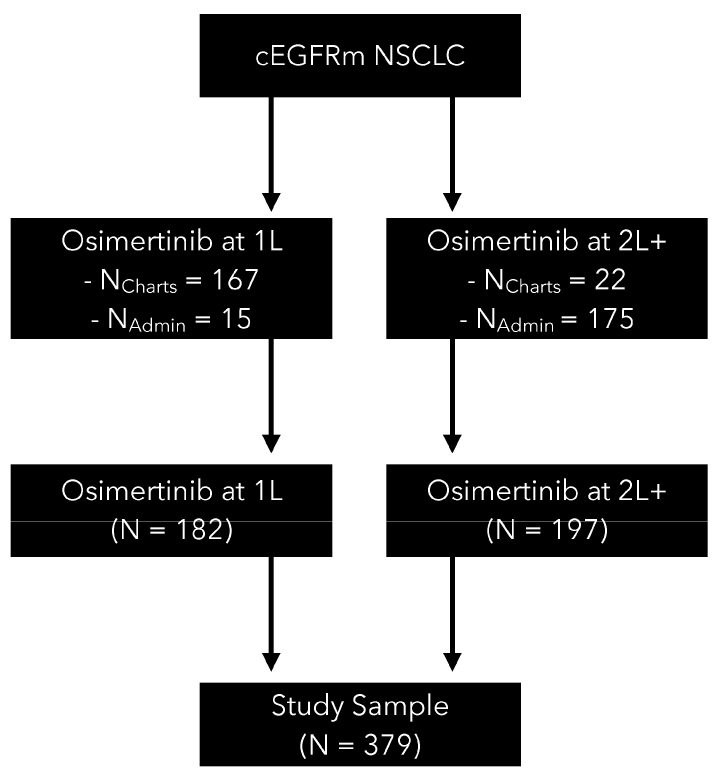
Flowchart for the final analytic sample of cEGFRm NSCLC patients receiving osimertinib in Alberta, Canada.

Our study identified 379 advanced/metastatic NSCLC patients with cEGFRm treated with osimertinib in Alberta, Canada with 182 (48%) and 197 (52%) receiving osimertinib in the 1L and 2L+ settings, respectively (Figure 1). Patient characteristics of the advanced/metastatic NSCLC patients with cEGFRm treated with osimertinib are reported in Table 1. In our study, the median follow-up for the overall cohort since the initiation of osimertinib was 15.7 months. The median follow-up since the initiation of osimertinib for those receiving it in 1L and 2L+ was 15.0 months and 17.0 months, respectively. For the post-osimertinib cohort, the median follow-up was 6.9 months since the initiation of post-osimertinib treatment and was 6.5 months and 7.3 months after 1L and 2L+ osimertinib, respectively. In the overall cohort, 68.3% of patients were women. The mean age at diagnosis was 66 years, 90.8% were treated at an academic facility, and 79.6% had stage IV disease at diagnosis. In the post-osimertinib cohort, 59.3% of patients were women. The mean age at diagnosis was 61 years and 77.6% were stage IV disease at diagnosis. Of the 86 patients in the post-osimertinib cohort, 26 (30.2%) and 60 (69.8%) previously received osimertinib in the 1L or 2L+ settings, respectively.

### 3.2. Overall Cohort: OS and TTNTD

**Figure 2:**
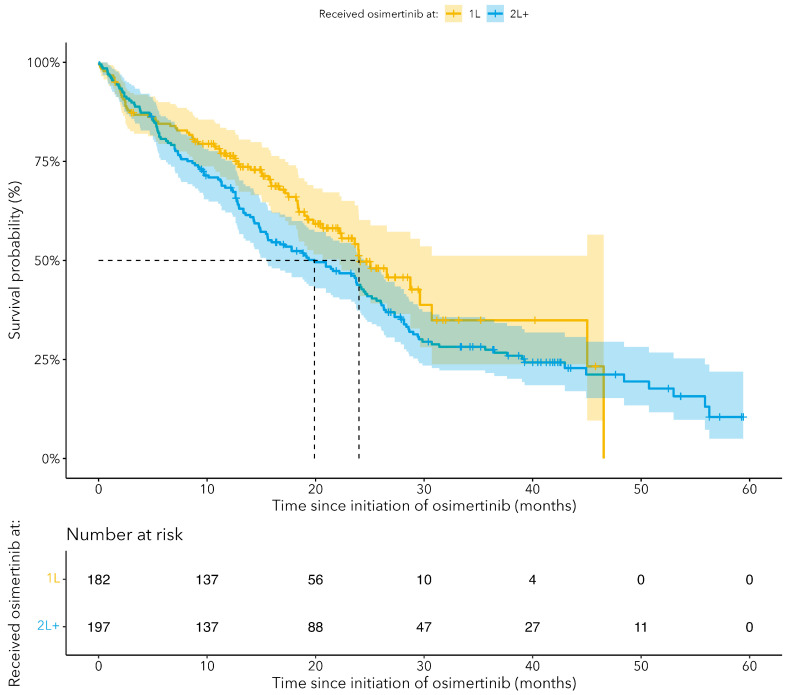
Kaplan–Meier curves for overall survival (OS) since the start of treatment with osimterinib, stratified by line of therapy when osimertinib was received (1L or 2L+).

**Figure 3:**
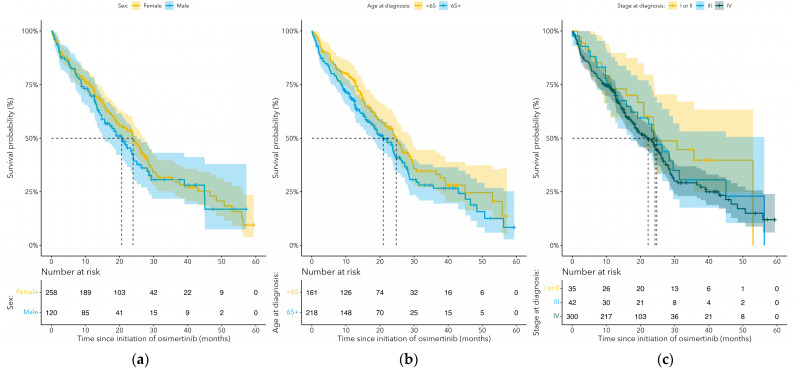
Kaplan–Meier curves for overall survival (OS) since the start of osimertinib for cEGFRm NSCLC patients treated with osimertinib at any line of therapy, stratified by (**a**) sex, (**b**) age at diagnosis, and (**c**) cancer stage at diagnosis.

The median OS for cEGFRm NSCLC patients treated with osimertinib in the 1L setting was 24.0 months (95% CI: 22.2–NA) and 19.9 months (95% CI: 15.6–24.4) for patients treated with osimertinib in the 2L+ settings (Figure 2, Table 2). Survival rates at 6 months, 1 year and 2 years were 84.5%, 76.4%, and 49.7%, respectively, in patients treated with 1L osimertinib and 80.7%, 68.3%, 43.9%, respectively, in patients treated with 2L+ osimertinib. Rates were similar among women and men, lower for patients aged 65 and older, and lower for advanced stage disease (Figure 3). The median TTNTD for cEGFRm NSCLC patients receiving 1L osimertinib was 19.5 months (95% CI: 16.7–26.6) and 14.9 months (95% CI: 12.2–20.9) for patients receiving 2L+ osimertinib (Table 2). The median TTNTD was 18.8 months (95% CI: 16.7–24.4) for women and 15.7 months (95% CI: 12.4–21.8) for men (Table 2).

### 3.3. Post-Osimertinib Cohort: OS and TTNTD

**Figure 4:**
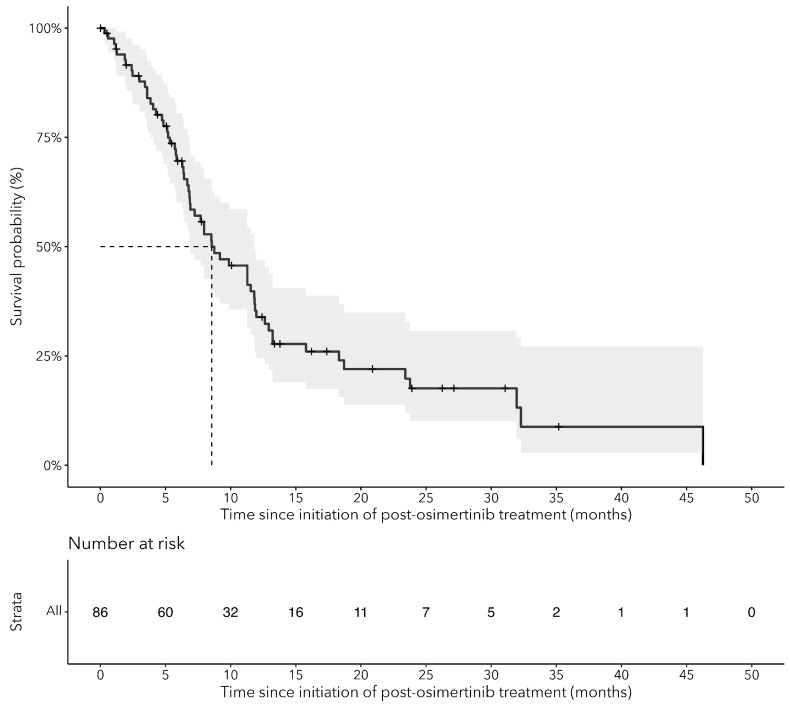
Kaplan–Meier curves for overall survival (OS) since the start of osimertinib for cEGFRm NSCLC patients who received treatment after osimertinib (post-osimertinib).

**Figure 5:**
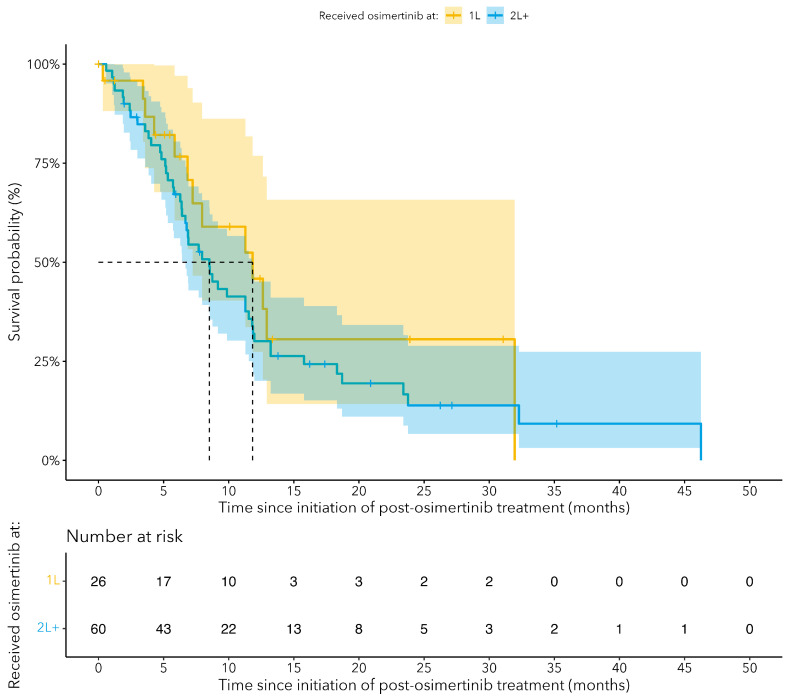
Kaplan–Meier curves for overall survival (OS) since the start of post-osimertinib therapy for cEGFRm NSCLC patients who received treatment after osimertinib, stratified by the line of therapy at which patients received osimertinib.

**Figure 6:**
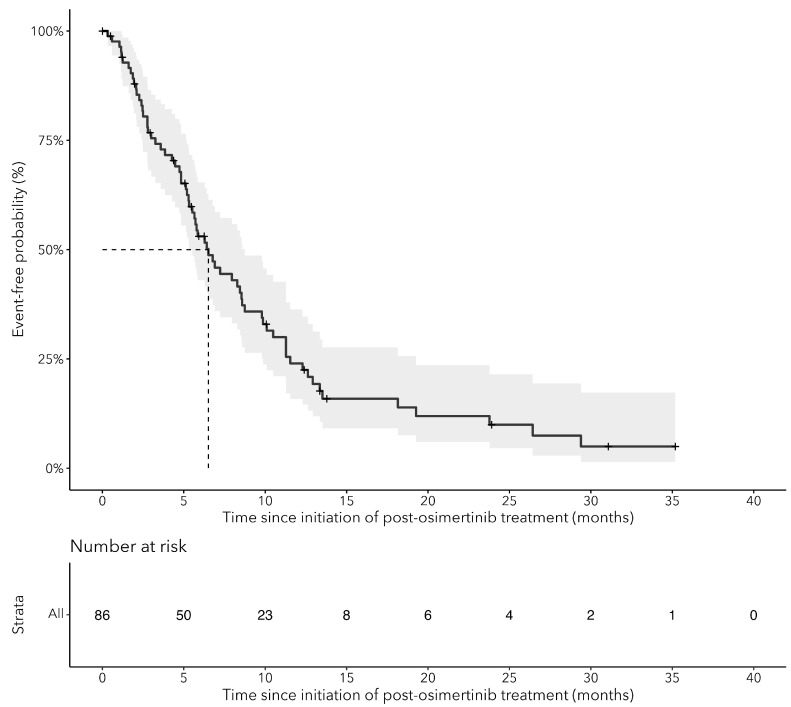
Kaplan–Meier curves for time-to-next-treatment or death (TTNTD) from the initiation of therapy after osimertinib for cEGFRm NSCLC patients who received treatment after osimertinib (post-osimertinib).

Median OS from the initiation of the subsequent therapy after osimertinib was 8.6 months (95% CI: 6.9–11.8) (Figure 4). We observed a rapid drop off in survival after 8 months, with less than 25.0% alive at 24 months (Figure 4). When stratified by line of osimertinib therapy, OS was 11.8 months (95% CI: 7.2–NA) for patients who received osimertinib as the 1L and 8.5 months (95% CI: 6.7–11.8) for those who received osimertinib as the 2L (Figure 5). The median TTNTD for the post-osimertinib cohort was 6.5 months (95% CI: 5.3–8.8) (Figure 6). When stratified by line of osimertinib therapy, the median TTNTD was 11.3 months (95% CI: 5.9–NA) for 1L and 5.7 months (95% CI: 4.8–8.6) for 2L.

### 3.4. Healthcare Resource Utilization

In the overall osimertinib cohort, we observed an average of 8.9 ambulatory care visits and 7.5 non-emergency care visits in the first year of follow-up. The mean number of hospitalizations per person was 0.7 with an average duration of 6.4 days hospitalized (Table 3). We observed an average of 5 or fewer ambulatory care visits per year from the second year onwards with a mean number of hospitalizations of 0.5 per person for an average number of days hospitalized between 4.4 to 5.0 in years 2 and 3 (Table 3). The average number of hospitalizations was similar in the post-osimertinib cohort when compared to the overall cohort, although the average number of days hospitalized was lower (3.4 days vs. 6.4 days).

### 3.5. Treatment Patterns

**Figure 7:**
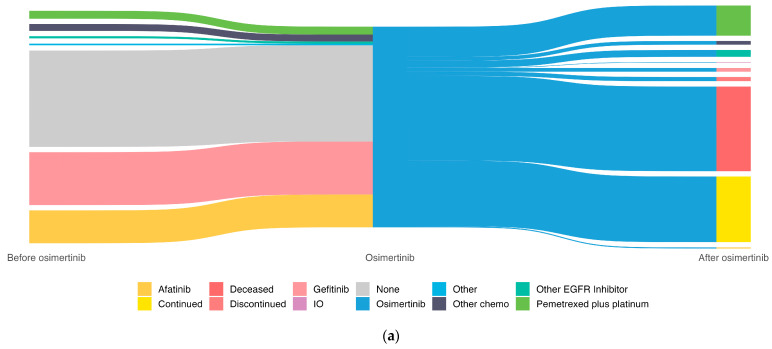

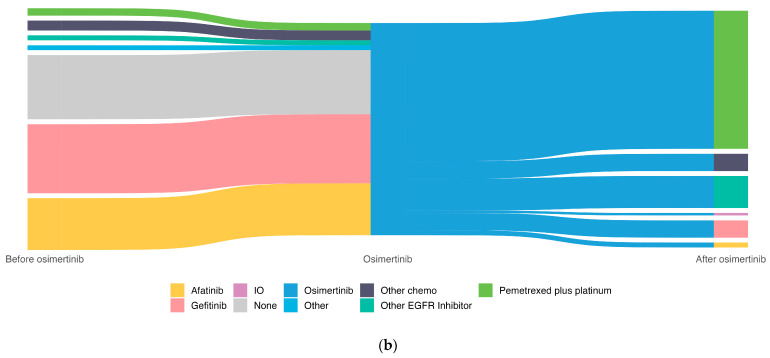
Sankey diagram for cEGFRm NSCLC treatments received immediately before or after osimertinib for the (**a**) overall cohort (N = 379) and (**b**) post-Osi cohort (N = 86).

In this study, 182 patients received 1L treatment with osimertinib and 197 patients underwent other therapies prior to osimertinib (100 gefitinib, 62 afatinib, 15 pemetrexed plus platinum, 13 other chemotherapies, and 7 other treatments) (Figure 7a). There were 293 individuals who did not or had not yet received subsequent therapies after receiving osimertinib (Figure 7a). Reasons for not receiving a post-osimertinib treatment included death (n = 160, 54.6%), patients were still on osimertinib at data cut-off (n = 124, 42.3%), and nine (3.1%) patients were alive and discontinued osimertinib but did not receive post-osimertinib treatment. Among individuals who received 1L Osimertinib (n = 182) but did not receive subsequent treatment, 86 were still on osimertinib (47.3%), 63 were deceased (34.6%), and 6 were alive and discontinued treatment and had not yet received a post-osimertinib treatment (3.3%). Among those receiving 2L+ osimertinib (n = 197), 97 were deceased (49.2%), 38 were still on treatment (19.3%), and 2 were alive and discontinued treatment without receiving a post-osimertinib treatment (1.0%). Individuals receiving 1L osimertinib were, on average, more recently diagnosed with a shorter follow-up (median of 1.4 years since diagnosis) than those on 2L+ osimertinib (median of 3.6 years since diagnosis). For those who proceeded to post-osimertinib treatment (N = 86), 56 individuals received pemetrexed plus platinum, 13 received another EGFR inhibitor, and 17 received other treatments (Figure 7b).

## 4. Discussion

These data represent one of the few large population-based studies to characterize the experience with osimertinib among advanced NSCLC patients harboring a cEGFRm. The measured baseline characteristics of our overall cohort of 379 patients treated with osimertinib were generally comparable to participants in the previous FLAURA (N = 279) and AURA3 (N = 279) clinical trials [12,13]. Our study consisted of 32% men (68% women) compared to 36% and 38% men in the FLAURA and AURA3 studies, respectively [12,13]. The median ages in FLAURA and AURA3 were 64 and 62 years as compared to an average age of 64 years in our overall cohort [12,13]. Our cohort had similar proportions of patients with adverse prognostic characteristics, such as brain metastases at baseline (21.1% vs. 19% in FLAURA). With regards to known prognostic factors, such as mutational status of the primary tumor, 237 (62.5%) and 137 (36.1%) of individuals in our study had Exon 19 deletions or L858R mutations, respectively (five individuals’ specific EGFR mutations were unspecified). Similar proportions of mutation status were found in FLAURA, with 175 (63.2%) individuals with Exon 19 deletions and 104 (37.5%) with L858R mutations.

### 4.1. Overall Cohort

The survival outcomes observed in patients receiving osimertinib in the current real-world study differed from those reported in the pivotal FLAURA (1L osimertinib) and AURA3 (2L osimertinib) trials [12,13]. The median OS for FLAURA was 38.6 months (95% CI: 34.5–41.8) compared to 24.0 months in our study [12]. In contrast, the median OS for 2L osimertinib was 20 months in our study, which was similar to AURA3 [13]. In these clinical trials, the median PFS was 18.9 (95% CI: 15.2–21.4) and 10.1 (95% CI: 8.3–12.3) months in 1L and 2L osimertinib use, respectively. Our study showed a median TTNTD of 19.5 months in 1L osimertinib and 14.9 months in 2L+ osimertinib. These differences are not surprising, given the non-selective, population-based inclusion of real-world patients in this analysis who often differ from those eligible to participate in clinical trials. The survival outcomes observed in the real-world setting underscore an unmet need for more effective 1L treatments in cEGFRm patients. The FLAURA2 study found that PFS was substantially longer for patients treated with osimertinib in combination with chemotherapy (25.5 months) compared to osimertinib alone (16.7 months) [11]. Preliminary results from the MARIPOSA study found a PFS advantage (24 versus 16 months) for amivantamab plus lazertinib as compared to osimertinib in the 1L setting; mature OS data are pending [23]. Of note, our study also demonstrated that the majority of patients (54.6%) did not proceed to subsequent therapies due to death, underscoring the importance of maximizing treatment benefit in the 1L setting. 

The subgroup analyses from FLAURA [12] suggested that patients with non-Asian ancestry may benefit more from osimertinib than those from Asia (HR 0.33 vs. 0.55, respectively). We were unable to capture detailed demographic data in this study due to logistical barriers, but understanding this balance of ethnicities will be critical to further our understanding of the driving forces for poorer survival in our cohort compared to FLAURA, separate from the obvious imbalances (age, ECOG, burden of disease, requirements for CNS control, etc.) between a trial-eligible and real-world population.

Management of disease progression on osimertinib is challenged by the plethora of on and off target resistance mechanisms driving the disease. Post-osimertinib treatment selection is challenging due to a lack of a predominant mechanism of resistance. Optimal treatment selection is critical to prolong survival of the patients who are able to receive further systemic therapy post progression on osimertinib. The lack of availability, in Alberta, to provincially funded next generation sequencing upon osimertinib progression (ctDNA or tissue) may have contributed to the shortened survival in this cohort of real-world treated patients.

Management of oligoprogressive disease requires a multimodal approach that may include local ablative therapies such as SBRT or SRS with high (80–90% at 1 year) locoregional disease control rates, permitting patients to stay on osimertinib post progression. Lack of availability of these local therapies in the 9.2% of patients in this cohort treated at community centers may have contributed to the inferior overall survival in the cohort as a whole. 

As the treating community waits for the MARIPOSA-2 [15] regimen amivantamab plus chemotherapy to become available in socialized medicine systems such as Canada, for patients progressing on osimertinib, we are left with platinum doublet chemotherapy (usually with pemetrexed) for eligible patients requiring a 2L therapy at present. Other jurisdictions are able to gain ongoing exposure to targeted therapies in this context after elucidating the mechanism of resistance, e.g., MET driven approaches with MET dysregulated disease. Neither of these approaches are available in Alberta at present, potentially contributing to the outcomes noted.

### 4.2. Post-Osimertinib Cohort

Patients who progress on osimertinib and receive a subsequent treatment have generally poor OS outcomes (~9 months median survival) and this is observed regardless of whether osimertinib was received in the 1L (11.8 months) or 2L+ (8.5 months) setting. The median TTNTD for post-osimertinib patients was short (6.5 months) and only 24% of post-osimertinib patients were event-free (no progression or death) at 1 year of follow-up. However, these findings differed based on the timing of receipt of osimertinib: median TTNTD of 11.3 months and 47.6% event-free at 1 year when osimertinib is given as 1L; median TTNTD of 5.7 months and 16.7% event-free at 1 year when osimertinib is given as 2L+. Attrition appears to be substantial with many patients (37.9% and 50.2% for 1L and 2L+ osimertinib) failing to proceed to subsequent lines of therapy after osimertinib, primarily due to death. This highlights the need for more effective treatment options post-osimertinib that can help to improve overall survival and reduce disease progression. Importantly, these findings should be interpreted with caution since patients who were unsuitable to receive subsequent therapies likely differed greatly from those who were well enough to undergo more treatments. 

### 4.3. Treatment Patterns and Healthcare Resource Utilization

In our study, a large proportion (74.9%) of patients either died (42.2%) or were still on osimertinib (32.7%) and therefore did not or had not yet received a subsequent treatment (i.e., post-Osi). In a context where patients may well only receive one line of therapy, as outlined here, it is important to optimize the benefit provided by the treatment chosen. This finding is related to the fact that the majority of the patients receiving 1L osimertinib were more recently diagnosed, due to the dates of approvals and funding for 1L osimertinib use in Alberta (and Canada as a whole), with a resultant shorter median follow-up. 

Among those treated with osimertinib, HCRU was highest in the first year after diagnosis. Few (≤5.5) ambulatory encounters occurred per year after year 2; however, these patients on average had ≥4 emergency department visits per year, suggesting persistent use of healthcare resources. Unfortunately, the data are not granular enough to determine the reasons for these hospitalizations or ER visits. HCRU was generally higher among those who progressed to receive post-osimertinib treatments compared to the overall cohort, especially in later years where more non-emergency visits were observed. While osimertinib is considered generally well tolerated by patients, the persistent number of non-emergency encounters and hospitalizations extending well beyond the first year, alongside the low number of planned ambulatory encounters, indicates that the morbidity burden of this disease, even when treated with osimertinib, remains significant and that there is an opportunity to minimize HCRU admissions and emergency room visits by potentially increasing ambulatory encounters with the novel therapeutic approaches previously mentioned.

### 4.4. Strengths and Limitations

The administrative data used in our study were population-based and captured nearly all eligible NSCLC cases for inclusion in our study. The quality of the systemic therapy data was also comprehensive, considering the short lag period between drug dispensing and data availability. Further, the findings of our study are expected to generalize well to other provinces, as has been shown in previous studies using similar data [24,27,28,29,30]. Our study has several limitations. First, the use of routinely-collected administrative data means that some important clinical data were not available for assessment, such as ECOG and ethnicity. Consistent with the RWE literature, we used TTNTD as a surrogate for PFS. However, discontinuation of therapy may occur for a variety of reasons (e.g., patient choice, toxicity, progression) that are not routinely collected in administrative data. Additionally, patients experiencing low-volume oligoprogression can be treated with local ablative therapies to allow the continuation of osimertinib [31]. Thus, this may differ from the trial definition of PFS (mandated on clinical or radiological grounds). Our study also included some patients who had early-stage NSCLC at the time of diagnosis but later progressed to metastatic disease during follow-up. The specific timing of this disease progression is not captured in administrative data and inconsistently recorded in chart data. Finally, while our data contained some information on brain metastases, specific details on their outcomes were not available for comparison purposes.

## 5. Conclusions

We found that the overall survival times for real-world patients receiving osimertinib were less favorable than those observed in clinical trials. The attrition rate after osimertinib was substantial and high HCRU persisted across years. This study provides important real-world evidence on contemporary survival, treatment patterns, and healthcare use among cEGFRm NSCLC patients treated with osimertinib and suggests that further research efforts are needed to improve therapeutic options in both the 1L and 2L+ settings.

## Figures and Tables

**Table 1:** Patient and clinical characteristics for cEGFRm NSCLC treated with osimertinib in Alberta. Means and standard deviations are reported for continuous variables and frequencies and percentages are reported for categorical variables.

	Osimertinib (N = 379)	Post-Osimertinib (N = 86)
Follow-up time (months)	36.3 [30.2]	45.7 [30.8]
Source		
Administrative data	190 (50.1%)	61 (70.9%)
Chart abstraction	189 (49.9%)	25 (29.1%)
EGFR mutation status (%)		
Exon 19 deletion	237 (62.5%)	58 (67.4%)
L858R	137 (36.1%)	28 (32.6%)
Unspecified	NR	NR
Age at diagnosis	65.7 [12.8]	60.5 [12.5]
Sex		
Female	258 (68.3%)	51 (59.3%)
Male	120 (31.7%)	35 (40.7%)
Year of diagnosis		
<2015	30 (7.9%)	10 (11.6%)
2015	16 (4.2%)	NR
2016	31 (8.2%)	13 (15.1%)
2017	30 (7.9%)	NR
2018	47 (12.4%)	10 (11.6%)
2019	51 (13.5%)	13 (15.1%)
2020	19 (5.0%)	NR
2021	91 (24.0%)	16 (18.6%)
2022	64 (16.9%)	NR
Treatment facility		
Academic	344 (90.8%)	NR
Community	35 (9.2%)	NR
Stage at diagnosis		
I	26 (6.9%)	NR
II	NR	NR
III	42 (11.1%)	10 (11.8%)
IV	300 (79.6%)	66 (77.6%)
Charlson Index	0.7 [0.9]	0.6 [0.8]
Number of metastases		
0	55 (14.5%)	16 (18.6%)
1	65 (17.2%)	22 (25.6%)
2	32 (8.4%)	10 (11.6%)
3+	38 (10.0%)	13 (15.1%)
Unknown	189 (49.9%)	25 (29.1%)
Metastases location		
Lung	264 (69.7%)	58 (67.4%)
Bone	125 (33.3%)	33 (38.4%)
Liver	49 (12.9%)	16 (18.6%)
Brain	80 (21.1%)	16 (18.6%)
Other	41 (10.8%)	NR
Osimertinib discontinuation		
No	124 (32.7%)	NR
Yes	255 (67.3%)	NR
Osimertinib line		
1L	182 (48.0%)	26 (30.2%)
2L+	197 (52.0%)	60 (69.8%)
Vital status		
Alive	158 (41.7)	25 (29.1)
Deceased	221 (58.3)	61 (70.9)
Number of lines received	2.0 [1.2]	3.4 [1.2]

Notes: NR indicates data/cells that are not reportable/suppressed based on guidelines for identifiable data. Discontinuation of osimertinib can be for any reason, including death. For categorical variables with only two levels, both cells may be suppressed when one cell is below the reportable threshold. Stage is based on the stage at the time of diagnosis. If an individual’s diagnosis progressed to late stage they may be treated as such, but their stage at diagnosis remains the same.

**Table 2:** Median overall survival (OS) and median time-to-next-treatment or death (TTNTD) since the initiation of osimertinib for cEGFRm NSCLC patients treated with osimertinib in Alberta.

Strata	Median OS, Months	Strata
Sex		
Female	23.97 (19.9–27.3)	18.81 (16.7–24.4)
Male	20.65 (15.6–25.1)	15.68 (12.4–21.8)
Age group		
<65	24.76 (20.7–29.0)	17.19 (14.9–23.9)
65+	20.94 (17.8–24.6)	18.35 (15.0–23.3)
Cancer stage at diagnosis		
I or II	24.23 (20.9–NA)	20.94 (15.9–NA)
III	24.76 (17.2–NA)	22.39 (15.6–28.4)
IV	22.19 (18.4–25.6)	16.87 (14.9–21.8)
Osimertinib line		
1L	24.00 (22.2–NA)	19.50 (16.7–26.6)
2L+	19.89 (15.6–24.4)	14.93 (12.2–20.9)

Abbreviations: cEFGRm, common epidermal growth factor receptor mutation; NSCLC, non-small cell lung cancer; OS, overall survival; TTNTD, time-to-next-treatment or death.

**Table 3:** Healthcare resource utilization since the date of cancer diagnosis for up to five years of follow-up. Means are reported with total counts in parentheses. Patients were included if they were alive for any amount of time during the year of follow-up.

	Year 1	Year 2	Year 3	Year 4	Year 5
Ever treated with osimertinib (N = 379)					
No. patients alive	379	330	212	141	94
Inpatient hospitalizations	0.7 (274)	0.5 (158)	0.5 (88)	0.4 (54)	0.5 (40)
Days hospitalized	6.4 (2418)	4.4 (1354)	5.0 (972)	9.1 (1115)	6.2 (493)
Outpatient encounters	8.9 (3376)	5.4 (1635)	5.2 (1015)	4.7 (581)	4.8 (366)
Non-emergency	7.5 (2826)	4.2 (1279)	4.1 (803)	3.7 (488)	3.8 (293)
Emergency	1.5 (550)	1.2 (359)	1.1 (212)	1.0 (123)	1.0 (83)
Post-osimertinib (N = 86)					
No. patients alive	86	84	64	43	31
Inpatient hospitalizations	0.6 (53)	0.6 (45)	0.6 (35)	0.4 (17)	0.6 (15)
Days hospitalized	3.4 (293)	4.2 (337)	6.9 (419)	6.3 (259)	6.5 (168)
Outpatient encounters	9.1 (780)	5.8 (466)	6.6 (404)	4.8 (200)	5.7 (136)
Non-emergency	7.5 (641)	4.6 (364)	5.2 (316)	3.8 (160)	4.5 (113)
Emergency	1.6 (139)	1.3 (102)	1.4 (88)	1.0 (41)	1.1 (28)

Abbreviations: No., number.

## Data Availability

Individual-level data are not publicly available due to Canadian data privacy laws governing personal health information.
